# Clinical Simulation in Maternal Code: Healthcare Personnel Satisfaction and Participant Characteristics

**DOI:** 10.7759/cureus.111729

**Published:** 2026-06-29

**Authors:** Ana María Sandoval Moreno, Lesly Aurora Rivera Villalobos, Leonardo Prado Carvajal, Oscar Alejandro Carmona Hidalgo, Enrique Guillermo Alonso Maldonado, Marisela Camarena Hermosillo, Juan Luis Aceves Siordia, Fátima Daniela Barba Moreno, Guadalupe Deribet López Rodriguez, Yaocihuatl Castañeda Borrayo

**Affiliations:** 1 Clinical Simulation Center for Clinical and Surgical Excellence (CeSiECQ), Instituto Mexicano del Seguro Social (IMSS), Guadalajara, MEX

**Keywords:** clinical simulation, healthcare personnel, maternal safety, medical education, multidisciplinary training, obstetric emergencies, obstetric simulation, safety patient, satisfaction, simulation training

## Abstract

Introduction: Maternal mortality remains a priority public health issue and a sensitive indicator of healthcare system performance. Clinical simulation has been increasingly adopted as an educational strategy to prepare healthcare personnel for obstetric emergencies. This study aimed to describe the sociodemographic and occupational characteristics of participants in the Código Mater course and to evaluate their satisfaction and perceptions regarding a simulation-based educational strategy.

Materials and methods: An observational, descriptive, and cross-sectional study was conducted at the Centro de Simulación para la Excelencia Clínica y Quirúrgica (CeSiECQ), a regional simulation center of a public healthcare institution in western Mexico, between May 2022 and December 2025. A total of 577 healthcare professionals participated through non-probability convenience sampling. Sociodemographic and occupational characteristics were analyzed, and participant satisfaction was assessed using an institutional questionnaire consisting of closed-ended and open-ended items. Quantitative data were analyzed descriptively, and participant comments were examined through inductive thematic analysis.

Results: Participants were predominantly young adults aged 22-30 years (n = 494; 85.6%) and female (n = 388; 67.2%). Physicians represented the largest professional group, including operational physicians (n = 321; 55.6%), followed by medical residents (n = 87; 15.1%) and interns or social service trainees (n = 85; 14.7%). Most participants were affiliated with regional general hospitals (n = 204; 35.4%) and general zone hospitals (n = 124; 21.5%). Satisfaction levels were consistently high across all evaluated domains. Qualitative findings identified five major themes: quality of the educational experience, relevance of course content, instructor performance, perceived applicability to clinical practice, and course organization.

Conclusions: Participants reported highly favorable perceptions of the Código Mater simulation-based course. Within the scope of Kirkpatrick Level 1 (Reaction), the findings indicate a high level of acceptance of simulation-based training and highlight its perceived relevance and applicability for obstetric emergency education. Further studies incorporating objective educational and clinical outcomes are required to evaluate the broader impact of the program.

## Introduction

Maternal mortality continues to be a priority public health problem and a sensitive indicator of the performance of health systems. The World Health Organization reported that, in 2023, approximately 260,000 women died during pregnancy, childbirth, or the postpartum period, with about 92% of those deaths occurring in low- and middle-income countries; furthermore, most were preventable with timely care, skilled healthcare personnel, and effective access to quality services. Globally, the maternal mortality ratio was 197 per 100,000 live births in 2022 [1].

In Mexico, despite advances in reducing maternal mortality over the past few decades, it continues to represent a significant challenge for the health system, with considerable disparities among federal entities that reflect gaps in access, quality, and timeliness of obstetric care [2]. According to estimates from the Global Burden of Disease, the maternal mortality ratio in the country showed a sustained decrease between 1990 and 2015, followed by a slower rate of decline in subsequent years, evidencing a stagnation in the reduction of this indicator [2]. In addition, epidemiological analyses have documented that during the COVID-19 pandemic period, there was a significant increase in maternal mortality in Mexico, associated with the overload of the health system and delays in timely obstetric care [3]. 

Recent national reports continue to identify maternal mortality as a relevant public health concern in Mexico, with marked geographic variation across states and persistent challenges related to healthcare access, quality of care, and timely recognition and management of obstetric emergencies [2,4]. These findings underscore the need to strengthen institutional strategies aimed at improving the organization and delivery of obstetric emergency care.

Globally, the main causes of maternal death continue to be obstetric hemorrhage, responsible for approximately 27% of deaths, followed by hypertensive disorders of pregnancy, which contribute around 14%, as well as infections, unsafe abortion, and other direct complications of pregnancy, childbirth, and the postpartum period [2,4,5]. Most of these conditions are preventable through timely interventions, standardized clinical protocols, and adequate organization of health services [2]. In addition, it has been estimated that most maternal deaths are avoidable, highlighting persistent failures in the quality of obstetric care, particularly in early risk identification and prompt response to emergencies [1].

Various studies have shown that the occurrence of maternal death does not depend solely on the clinical condition of the patient but also on failures in the care system, particularly those described in the model of "three delays": delays in the decision to seek care, delays in accessing health services, and delays in providing adequate care within medical units [6]. These delays are associated with deficiencies in service organization, limitations in the training of healthcare personnel, delays in identifying warning signs, and failures in communication and coordination among the multidisciplinary team [7,8]. Furthermore, it has been documented that the quality of obstetric care, including adherence to clinical guidelines, availability of resources, and preparedness for obstetric emergencies, is a determining factor in reducing maternal morbidity and mortality [8,9]. In this context, structured institutional strategies and standardized protocols have become essential components of efforts aimed at improving obstetric care and patient safety [10].

In response to these limitations, strategies aimed at improving the management of obstetric emergencies have been developed through the implementation of organized response systems, including immediate activation codes and rapid response teams. These models, inspired by patient safety systems and crisis management, allow for the timely identification of severe complications, the activation of multidisciplinary teams, and the standardization of clinical interventions based on protocols [11,12]. In the obstetric context, these strategies have been associated with improvements in response times, team coordination, and adherence to clinical guidelines and have been linked to reductions in adverse maternal events [10,12]. In Mexico, Código Mater has been established as an institutional strategy designed to support the timely identification and management of obstetric emergencies through coordinated and standardized actions by healthcare personnel.

In similar studies, training in obstetric emergencies based on clinical simulation has shown significant impacts across multiple domains of healthcare personnel performance. In Mexico, the PRONTO program, implemented in 24 public hospitals, revealed that the educational intervention based on simulation was associated with a 21% reduction in the cesarean section rate (p = 0.005) and a 40% decrease in neonatal mortality at eight months post-intervention (incidence rate ratio (IRR) 0.60; 95% confidence interval (CI) 0.37-0.95), although without significant changes in overall maternal complications. Improvements were also observed in clinical processes, including increases of up to 25 percentage points in evidence-based practices during childbirth care [13,14].

In relation to individual learning and performance, previous studies have documented that obstetric simulation programs generate significant improvements in knowledge, technical and non-technical skills, and the self-efficacy of healthcare personnel, regardless of professional category. In a multicenter study in Mexico, training through simulation showed consistent increases in knowledge and self-efficacy among all participants, with improvements observed in both physicians and nursing personnel, in addition to a compliance rate of up to 65% of the institutional objectives proposed after the intervention [15].

Consistently, international systematic reviews have reported that clinical simulation in obstetric emergencies is associated with improvements in clinical performance, leadership, and teamwork, with significant increases in knowledge in the majority of studies analyzed and favorable changes in interdisciplinary communication [16].

These findings suggest that the main effects of clinical simulation are observed in intermediate variables such as learning, self-efficacy, adherence to protocols, and team coordination, while effects on hard clinical outcomes, such as maternal mortality, are more heterogeneous and depend on the institutional context, the intensity of training, and its integration into everyday clinical practice [13-16].

The objective of the present study was to describe the sociodemographic and occupational characteristics of healthcare personnel participating in the Código Mater course and to evaluate their satisfaction and perceptions regarding a simulation-based educational strategy. Given the descriptive and cross-sectional design, the study focused on participant characteristics and reactions to the educational experience (Kirkpatrick Level 1) rather than on the assessment of knowledge acquisition, skill performance, behavioral change, transfer of learning, or clinical outcomes.

## Materials and methods

An observational, descriptive, and cross-sectional study was conducted to characterize the sociodemographic and occupational characteristics of healthcare personnel participating in the Código Mater course, as well as to evaluate their satisfaction with a simulation-based educational strategy.

The study was conducted at the Centro de Simulación para la Excelencia Clínica y Quirúrgica (CeSiECQ), a regional referral simulation center belonging to a public social security institution in western Mexico, during the period from May 2022 to December 2025. The center conducts approximately 14-15 Código Mater courses annually and receives healthcare personnel from multiple Mexican states, including Aguascalientes, Baja California, Chihuahua, Coahuila, Colima, Durango, Guanajuato, Guerrero, Jalisco, Michoacán, Nayarit, Nuevo León, San Luis Potosí, Sinaloa, Sonora, Tamaulipas, and Zacatecas.

The study population consisted of healthcare personnel participating in the Código Mater course, including family physicians, emergency physicians, anesthesiologists, gynecologists-obstetricians, medical interns, residents, nursing staff, and other categories involved in the care of pregnant women. A total of 577 participants (n = 577) were included through non-probability convenience sampling based on course attendance during the study period. All healthcare personnel attending the course were invited to participate. A total of 577 individuals attended the course, and all completed the evaluation instruments, resulting in a response rate of 100%.

Inclusion criteria included participants who completed the sociodemographic questionnaire and the satisfaction evaluation (see Appendix). Because the electronic evaluation platform required completion of all questionnaire items before submission, no incomplete questionnaires were identified, and no participants were excluded from the final analysis.

Sociodemographic and occupational variables were analyzed, including age, sex, occupational category, affiliation, shift, specialty, and academic degree. The primary study variable was participant satisfaction with the Código Mater course.

Satisfaction was assessed using an institutional educational evaluation questionnaire routinely employed in the Código Mater course. The instrument consisted of eight closed-ended items distributed across domains related to overall course satisfaction, perceived contribution to learning, confidence and self-assurance in learning, adequacy of facilities and resources, interest in future simulation activities, instructor feedback, instructor content mastery, and facilities hygiene and conditions. Most items were answered using dichotomous response options (yes/no), whereas some items included categorical response options reflecting the level of agreement or satisfaction. In addition, the questionnaire included one open-ended question that allowed participants to provide comments and suggestions regarding the educational experience. The questionnaire was originally developed for institutional educational quality-assurance purposes and had not undergone formal psychometric validation, pilot testing, or reliability assessment. Quantitative responses were analyzed descriptively, whereas qualitative responses were subjected to thematic analysis. This approach allowed the integration of quantitative and qualitative information corresponding to Level 1 (Reaction) of Kirkpatrick’s evaluation model [16].

The intervention consisted of the implementation of the institutional Código Mater course, designed as a comprehensive simulation-based educational strategy. The program lasted eight hours and was structured into progressive theoretical-practical modules that included adult basic life support, endotracheal intubation, obstetric triage, arrhythmia management, and fluid therapy, as well as a specialized module on obstetric hemorrhage integrating management with blood products, uterotonics, vasoactive agents, and hemorrhage control techniques, including compressive sutures and intrauterine balloon placement. The management of obstetric sepsis was also included among the critical clinical scenarios. Throughout the study period (2022-2025), the course maintained standardized learning objectives, content, simulation scenarios, and evaluation procedures. Although instructors rotated between courses, all were specialists in Obstetrics and Gynecology with experience in obstetric emergency training and clinical simulation.

The central phase of the course involved the implementation of Código Mater clinical simulation scenarios in which high-complexity situations such as obstetric hemorrhage, preeclampsia-eclampsia, sepsis, and respiratory failure were recreated, allowing activation of multidisciplinary teams, real-time decision-making, and application of standardized care protocols. These activities were conducted using high-fidelity simulators, telesimulation, and simulated clinical environments replicating hospital settings such as operating rooms, resuscitation areas, and inpatient units, promoting the integration of technical and non-technical skills, including communication, leadership, and teamwork, fundamental elements of patient safety widely described in the clinical simulation literature [17,18,19].

For statistical analysis, descriptive statistics were used, including measures of central tendency and dispersion for quantitative variables and frequencies and percentages for categorical variables. No inferential analyses were performed because of the descriptive nature of the study. The analysis was conducted using IBM SPSS Statistics version 25 (IBM Corp., Armonk, NY, USA) and Microsoft Excel (Microsoft Corp., USA).

Additionally, open-ended comments were analyzed using an inductive thematic analysis approach. Five researchers independently reviewed and coded participant comments. Codes were subsequently compared and discussed until consensus was reached regarding the final thematic categories. The analysis focused on identifying recurring themes related to the educational experience, content relevance, instructor performance, perceived applicability to clinical practice, course organization, and suggestions for improvement. Formal inter-rater agreement statistics were not calculated. This analysis complemented the quantitative findings and enriched the interpretation of participants’ perceptions of the educational strategy.

The study and the analyzed information were derived from a protocol approved by the Research Ethics Committee and Local Health Research Committee of the Instituto Mexicano del Seguro Social (IMSS), Jalisco, Mexico. The Código Mater course was an institutional educational activity that predated the present investigation. The approval identified by registration number R-2025-1306-245 specifically authorized the analysis and publication of educational data generated through this institutional program. No additional interventions or data collection procedures were performed for research purposes. Confidentiality was ensured through the use of anonymized databases without direct participant identification, in accordance with the principles of the Declaration of Helsinki [20] and the currently applicable national regulations governing health research [21].

## Results

**Figure 1:**
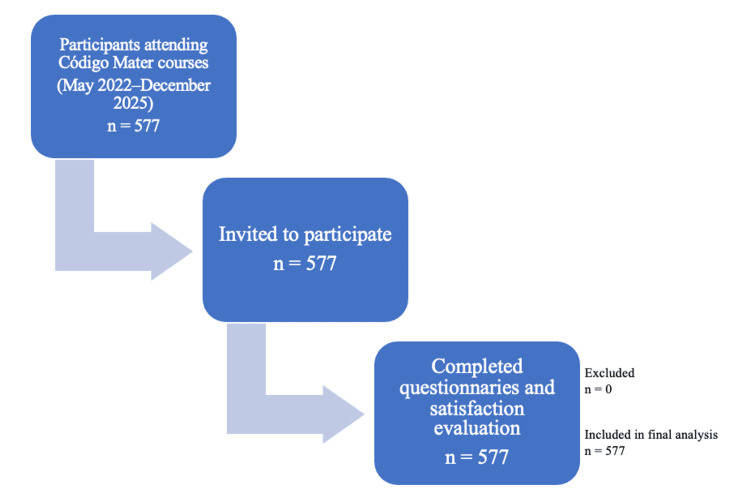
Flow diagram of participant selection and inclusion in the study. Source: Own elaboration based on participant records from the Código Mater course conducted at the Centro de Simulación para la Excelencia Clínica y Quirúrgica (CeSiECQ), May 2022–December 2025.

A total of 577 participants (n = 577) were included in the analysis of sociodemographic, occupational, and satisfaction variables. The study population consisted of healthcare personnel from diverse professional categories involved in the care of pregnant women, including physicians, nursing personnel, medical trainees, and other healthcare professionals. All participants who attended the Código Mater course during the study period completed the evaluation instruments and were included in the final analysis. The descriptive analysis revealed variability across sociodemographic and occupational characteristics, particularly with respect to age, occupational category, institutional affiliation, and work shift. The results are presented below according to the characteristics of the study population and the distribution of the analyzed variables (Figure 1).

Sociodemographic characteristics of the participants

Table 1 summarizes the sociodemographic characteristics of the study population (n = 577). Most participants were young adults, with the 22-30-year age group representing the largest proportion of the sample (n = 494; 85.6%). The 31-40-year age group included 76 participants (13.2%), whereas only seven participants (1.2%) were between 41 and 50 years of age. The predominance of younger participants is likely related to the substantial representation of medical interns, residents, and recently graduated healthcare personnel attending the course during the study period.

**Table 1:** Sociodemographic characteristics of the participants (n = 577). Source: Own elaboration based on the sociodemographic questionnaire.

Variable	Frequency	Percentage
Age (years)		
22–30	494	85.6
31–40	76	13.2
41–50	7	1.2
Sex		
Male	189	32.8
Female	388	67.2

Regarding sex distribution, 388 participants (67.2%) were female and 189 (32.8%) were male.

Occupational category of the participants

Table 2 presents the distribution of participants according to occupational category (n = 577). Physicians constituted the largest professional group, accounting for 346 participants (59.9%), including 321 operational physicians (55.6%) and 25 department heads (4.3%). Among operational physicians, obstetrician-gynecologists represented the largest subgroup (n = 156; 27.0%), followed by general physicians (n = 79; 13.7%), emergency physicians (n = 43; 7.5%), and anesthesiologists (n = 23; 4.0%). Other physician specialties were represented by smaller numbers of participants.

**Table 2:** Occupational category of the participants (n = 577) Source: Own elaboration based on the sociodemographic questionnaire.

Job category	Frequency	Percentage
Department head physicians	25	4.3
Obstetrician-gynecologists	156	27.0
General physicians	79	13.7
Emergency physicians	43	7.5
Anesthesiologists	23	4.0
Family physicians	5	0.9
Maternal-fetal medicine specialists	4	0.7
General surgeons	3	0.5
Pediatricians	3	0.5
Intensivists	2	0.3
Internists	1	0.2
Pediatric anesthesiologists	1	0.2
Urologists	1	0.2
General nurses	30	5.2
Surgical nurses	7	1.2
Nursing interns	5	0.9
Specialist nurses	4	0.7
Pediatric nurses	4	0.7
Head nurses	3	0.5
Intensive care nurses	2	0.3
Nursing technicians	2	0.3
Public health nurses	1	0.2
Administrative nurses	1	0.2
Medical residents	87	15.1
Interns	80	13.9
Medical social services trainees	5	0.9

Nursing personnel accounted for 59 participants (10.2%), with general nurses representing the largest subgroup (n = 30; 5.2%), followed by surgical nurses (n = 7; 1.2%) and nursing interns (n = 5; 0.9%). The remaining nursing categories individually represented less than 1% of the study population.

Medical specialty residents comprised 87 participants (15.1%), whereas medical interns and social service trainees represented 85 participants (14.7%), including 80 medical interns (13.9%) and five social service trainees (0.9%).

Institutional unit of affiliation

Table 3 presents the distribution of participants according to their institutional unit of affiliation. Regional General Hospitals (HGR) accounted for the largest proportion of participants, with 204 individuals (35.4%), followed by General Zone Hospitals (HGZ) with 124 participants (21.5%) and General Zone Hospitals with Family Medicine Units (HGZMF) with 92 participants (15.9%). Family Medicine Units (UMF), representing the first level of care, contributed 69 participants (12.0%).

**Table 3:** Institutional unit of affiliation of the participants (n = 577). Source: Own elaboration based on institutional affiliation records. *CMNO: Centro Médico Nacional de Occidente (National Medical Center of the West)

Unit type	Frequency	Percentage
Family Medicine Units (UMF)	69	12.0
General Zone Hospitals (HGZ)	124	21.5
Regional General Hospitals (HGR)	204	35.4
General Zone Hospitals with Family Medicine Units (HGZMF)	92	15.9
High-Specialty Medical Unit of Specialties, CMNO	33	5.7
High-Specialty Medical Unit of Gynecology and Obstetrics	54	9.4
High-Specialty Medical Unit of Pediatrics	1	0.2

High-specialty medical units accounted for a smaller proportion of attendees. The High Specialty Medical Unit of Gynecology and Obstetrics contributed 54 participants (9.4%), followed by the High Specialty Medical Unit of Specialties of the National Medical Center of the West (CMNO) with 33 participants (5.7%). The High Specialty Medical Unit of Pediatrics contributed one participant (0.2%).

This distribution reflects participation patterns among healthcare personnel attending the Código Mater course and should not be interpreted as representative of the overall workforce distribution within the institution. The predominance of participants from regional and general hospitals may be related to the frequent involvement of these facilities in the initial recognition and management of obstetric emergencies, whereas high specialty units contributed fewer participants proportionally because of their more specific referral roles within the healthcare network.

Work shift distribution

The morning shift accounted for the largest proportion of participants, with 317 individuals (55.0%), followed by the mixed shift with 136 participants (23.6%). The evening shift included 59 participants (10.2%), whereas the night shift comprised 51 participants (8.8%). The accumulated shift represented the smallest group, with 13 participants (2.4%) (Table 4). 

**Table 4:** Distribution of participants according to work shift (n = 577) Source: Own elaboration based on institutional affiliation records Note: The mixed shift corresponds to personnel with rotating or variable schedules involving coverage of different shifts (morning, evening, and/or night) according to service requirements.

Work shift	Frequency	Percentage
Morning shift	317	55.0
Evening shift	59	10.2
Night shift	51	8.8
Mixed shift	136	23.6
Accumulated shift	13	2.4

Participant satisfaction

Participant satisfaction with the Código Mater simulation course was exceptionally high across all evaluated domains. Overall course satisfaction was reported by 99.8% of participants (576/577). All participants indicated that the course contributed to their learning and enhanced their confidence in managing clinical scenarios (100.0% each).

The educational environment was also highly rated, with 99.7% of participants considering the facilities and available resources adequate for learning. Likewise, 99.8% expressed interest in attending additional simulation-based training activities, reflecting a strong acceptance of simulation as an educational strategy.

Regarding instructor performance, all participants reported receiving feedback from instructors, and 97.6% perceived extensive mastery of course content among faculty members. Furthermore, 99.0% rated the facilities as completely hygienic, highlighting the favorable conditions of the simulation center.

Overall, these findings demonstrate a high level of participant satisfaction with the educational experience, instructor performance, learning environment, and perceived educational value of the simulation-based Código Mater program (Table 5).

**Table 5:** Participant satisfaction with the Código Mater simulation-based training program (N = 577). Source: Institutional satisfaction questionnaires administered to participants attending the Código Mater simulation course at the Centro de Simulación para la Excelencia Clínica y Quirúrgica (CeSiECQ). Note: Only favorable response categories are presented in the table. Unfavorable responses were infrequent and are described in the text when applicable.

Evaluated factor	Response category	n (%)
Overall course satisfaction	Satisfied	576 (99.8)
Contribution to learning	Yes	577 (100.0)
Contribution to confidence and self-assurance in learning	Yes	577 (100.0)
Adequacy of facilities and resources	Adequate	575 (99.7)
Interest in attending additional simulation activities	Yes	576 (99.8)
Feedback received from instructors	Yes	577 (100.0)
Instructor content mastery	Extensive mastery	563 (97.6)
Facilities, hygiene, and conditions	Completely hygienic	571 (99.0)

Qualitative findings

Open-ended comments were analyzed using an inductive thematic approach. Five researchers independently reviewed participant responses and reached consensus regarding the final thematic categories. Five major themes emerged from the analysis: quality of the educational experience, relevance of course content, instructor performance, perceived applicability to clinical practice, and course organization. Table 6 summarizes the themes identified through the qualitative analysis of participants’ open-ended comments.

**Table 6:** Themes identified through qualitative analysis of open-ended participant comments. Source: Qualitative thematic analysis of open-ended comments obtained from institutional satisfaction questionnaires administered to participants attending the Código Mater simulation course at the Centro de Simulación para la Excelencia Clínica y Quirúrgica (CeSiECQ). Note: Themes were identified through inductive thematic analysis. Five researchers independently reviewed and coded participant comments, followed by consensus meetings to establish the final thematic categories.

Theme	Description	Representative quotation
Quality of the educational experience	Participants described the course as dynamic, engaging, and useful for integrating theoretical knowledge with practical activities in a safe learning environment.	“It was an excellent course, complete and dynamic.”
Relevance of course content	Participants considered the topics highly relevant and applicable to real obstetric emergencies encountered in clinical practice.	“Very important topics for the management of obstetric emergencies.”
Instructor performance	Participants highlighted instructor expertise, clarity of explanations, professionalism, and constructive feedback during practical activities.	“Highly trained instructors with clear explanations.”
Perceived applicability to clinical practice	Participants reported that the course reinforced protocols, improved preparedness, and increased confidence in managing obstetric emergencies.	“I feel more prepared to face an obstetric emergency.”
Course organization and suggestions for improvement	Although participants positively evaluated course organization, some recommended extending course duration and providing preparatory materials before attendance.	“The time allocated to some topics could be extended.”

Quality of the educational experience

The participants described the educational experience as highly favorable, emphasizing the value of the simulation-based approach for integrating theoretical knowledge with practical activities in a safe and controlled learning environment. Comments frequently highlighted the dynamic nature of the course and the opportunity to actively participate in realistic clinical scenarios.

“It was an excellent course, complete and dynamic.”

“The simulation scenarios facilitated learning.”

Relevance of the course content

Participants positively evaluated the relevance of the course content, particularly its relationship to real-world obstetric emergencies. Many respondents considered the topics directly applicable to situations commonly encountered in clinical practice.

“Very important topics for the management of obstetric emergencies.”

“Content suitable for daily practice.”

Instructor performance

Instructor performance was consistently viewed favorably. Participants highlighted the instructors’ expertise, teaching skills, clarity of explanations, and willingness to provide guidance and feedback during practical activities.

“Highly trained instructors with clear explanations.”

“Good guidance during practices.”

Perceived applicability to clinical practice

One of the most frequently reported themes was the perceived applicability of the educational experience to professional practice. Participants indicated that the course reinforced clinical protocols, increased awareness of obstetric emergencies, and strengthened their confidence in managing complex clinical situations.

“The things I learned are applicable in my daily medical practice.”

“I feel more prepared to face an obstetric emergency.”

Course organization and suggestions for improvement

Overall, the participants expressed positive opinions regarding course organization, structure, and sequencing of activities. Nevertheless, several respondents suggested extending the duration of the course, increasing the time allocated to simulation scenarios, and providing preparatory materials before attendance.

“Good organization of the course.”

“The time allocated to some topics could be extended.”

Overall, the qualitative findings demonstrated a predominantly favorable perception of the Código Mater simulation-based course. Participants highlighted the educational value of the experience, the relevance of the content, the quality of instruction, and the perceived applicability of learning to clinical practice. Suggestions for improvement primarily focused on extending course duration and increasing opportunities for practical simulation activities.

## Discussion

The present study allowed for the characterization of the sociodemographic and professional profile of the healthcare personnel participating in the Código Mater course, as well as the evaluation of their satisfaction regarding an educational strategy based on clinical simulation. The findings show a predominance of young personnel in training or in the early stages of their professional practice, as well as a broadly favorable perception of the training experience, particularly regarding its clinical applicability and the integration of competencies. 

Most participants were between 22 and 30 years of age (85.6%), suggesting that the Código Mater simulation-based course primarily reached healthcare personnel in training or in the early stages of professional development. Early exposure to simulation-based training in obstetric emergencies may contribute to strengthening preparedness, teamwork, and patient safety competencies throughout professional practice. Likewise, the participation of multiple professional categories, including operational physicians, residents, interns, and nursing personnel, reflects the interdisciplinary nature of obstetric emergency management and the need to strengthen teamwork in critical scenarios, consistent with the literature on clinical simulation [18].

In terms of institutional distribution, the greater involvement of second-level hospital units, particularly regional and zonal general hospitals, suggests that these strategies are being implemented in contexts where the burden of obstetric care is significant. This finding is consistent with the need to strengthen the problem-solving capacity in units that face a high volume of obstetric emergencies, which has been identified as a key factor in reducing maternal morbidity and mortality [10]. Although the study was conducted at a single simulation center, participants originated from multiple healthcare facilities and several Mexican states, providing diversity in professional backgrounds and institutional contexts. This broad participation enhances the contextual relevance of the findings and reflects the regional educational role of the simulation center.

Regarding participant satisfaction, the results showed favorable perceptions across all evaluated dimensions, particularly the quality of the training experience and the perceived applicability of learning. These findings are consistent with previous studies that have demonstrated that clinical simulation improves the perception of learning, self-efficacy, and the confidence of healthcare personnel in managing critical situations [15,16]. The perception of integration between theory and practice observed in this study aligns with evidence indicating that simulation facilitates meaningful learning through exposure to realistic clinical scenarios in safe environments [17].

The qualitative findings complemented the quantitative results and provided additional insight into participant perceptions. The convergence between quantitative satisfaction outcomes and qualitative themes strengthens the credibility of the findings. Participants not only reported high levels of satisfaction but also consistently described the educational experience as relevant, engaging, and applicable to their professional practice. This triangulation of findings provides a more comprehensive understanding of participant reactions to the Código Mater educational strategy.

Likewise, the positive evaluation of the instructors' performance and the feedback received during practical activities reinforces the role of structured debriefing as a central component of simulation-based education. Various studies have documented that the quality of debriefing directly influences the consolidation of learning and the transfer of competencies to clinical practice [19].

Regarding the relevance of the content, participants noted that topics addressed reflect real clinical situations, particularly in the context of obstetric emergencies. This finding is consistent with the PRONTO program's approach, in which contextualized simulation has been shown to improve adherence to evidence-based practices and optimize clinical processes during childbirth care [13,14].

In this sense, the alignment between educational content and the needs of the clinical environment constitutes a key element for the perceived educational value of training interventions. The applicability of learning emerged as one of the most relevant dimensions in the participants’ perception, who reported feeling better prepared to face obstetric emergencies. These results align with international evidence indicating that clinical simulation has a significant impact on intermediate variables such as confidence, teamwork, and clinical decision-making, factors that indirectly influence health outcomes [16]. However, as previously documented, the translation of these effects into improvements in hard clinical outcomes, such as maternal mortality, depends on multiple contextual factors and the sustained integration of training into daily practice [13].

The findings should be interpreted within the framework of Kirkpatrick Level 1 (Reaction), as the present study evaluated participant satisfaction and perceptions regarding the educational experience rather than objective measures of knowledge acquisition, skills performance, behavioral change, transfer of learning, or clinical outcomes. Therefore, the results should not be interpreted as evidence of educational effectiveness or direct improvements in clinical performance.

Regarding course organization, although the overall perception was favorable, some participants identified opportunities for improvement related to the distribution of time across modules. This finding suggests the need for adjustments in instructional design to optimize the duration and depth of content, particularly in topics involving greater clinical complexity.

The present study has several limitations that must be considered. First, its observational, descriptive, and cross-sectional design limits the possibility of establishing causal associations between the analyzed variables. Secondly, the use of non-probability convenience sampling restricts the generalization of the results to other populations or institutional contexts. Furthermore, participants were recruited exclusively from healthcare personnel attending the Código Mater course; therefore, the findings should not be interpreted as representative of all healthcare personnel within the institution or of the national healthcare workforce. Likewise, the evaluation of satisfaction was based on participants’ perceptions, which may be influenced by social desirability bias.

Additionally, satisfaction was assessed using an institutional questionnaire developed for educational quality-assurance purposes that had not undergone formal psychometric validation, pilot testing, or reliability assessment. Consequently, the findings should be interpreted as descriptive indicators of participant perceptions rather than measurements obtained through a validated evaluation instrument. Although five researchers independently reviewed and coded participant comments and subsequently reached consensus regarding the final thematic categories, formal measures of inter-rater reliability were not calculated. Therefore, the consistency of qualitative coding was established through consensus procedures rather than statistical agreement measures. Another limitation is the uniformly high satisfaction scores observed across most evaluated domains. This may indicate the presence of a ceiling effect, reducing the ability of the instrument to discriminate among different levels of participant satisfaction and potentially limiting variability in responses. Comparisons between groups and analyses of variables associated with satisfaction levels were not included, which limits the identification of factors potentially associated with participant perceptions of the course. Despite these limitations, the study provides relevant evidence regarding the sociodemographic and professional profile of healthcare personnel participating in the Código Mater course, as well as their perceptions of a simulation-based educational strategy. The findings demonstrate a high level of acceptance of the course and a favorable evaluation of its training components, supporting its relevance as an institutional strategy for obstetric emergency training.

Clinical simulation within the Código Mater program was perceived by participants as a relevant, applicable, and highly valued educational strategy for obstetric emergency training. Its implementation in institutional settings represents an opportunity to strengthen preparedness, teamwork, and confidence in the management of obstetric emergencies. However, future studies incorporating objective measures of learning, behavioral transfer, and clinical outcomes are necessary to determine the broader educational impact of the program.

## Conclusions

The Código Mater simulation-based training course was implemented in a predominantly young and multidisciplinary population, mainly composed of physicians, residents, interns, and nursing personnel, with most participants affiliated with second-level healthcare facilities. The participants reported high levels of satisfaction with the educational strategy, particularly regarding the quality of the training experience, the relevance of course content, instructor performance, and the perceived applicability of learning to clinical practice, especially in the management of obstetric emergencies.

Within the scope of Kirkpatrick Level 1 (Reaction), the findings indicate a highly favorable perception of the educational experience and a high level of acceptance of simulation-based training among healthcare personnel. The integration of quantitative satisfaction results and qualitative participant perceptions provides a comprehensive understanding of learner reactions to the Código Mater program.

Although the findings support the value of simulation as an educational strategy for obstetric emergency training, future studies should incorporate validated evaluation instruments and objective measures of knowledge acquisition, skills performance, behavioral transfer, and clinical outcomes to determine the broader educational impact of the program.

## Appendices

Sociodemographic and Occupational Questionnaire

The following variables were collected through the institutional participant registration form completed prior to attendance at the Código Mater course: 1. Age (years) 2. Gender • Female • Male • Other 3. Professional category • Physician • Nursing personnel • Other 4. Specific professional category 5. Participant type • Undergraduate trainee • Resident physician • Continuing education personnel • Administrative/confidence staff 6. Specialty 7. Highest academic degree attained • High school • Technical degree • Bachelor's degree • Specialty • Master's degree • Doctorate • Postdoctoral training • Other 8. Institutional unit of affiliation 9. Service of affiliation 10. State/Regional Delegation (OOAD) of affiliation 11. Work shift • Morning • Evening • Night • Mixed • Accumulated 12. Course attended 13. Previous attendance at the simulation center • Yes • No
 
